# Clinical report of cervical arthroplasty in management of spondylotic myelopathy in Chinese

**DOI:** 10.1186/1749-799X-1-13

**Published:** 2006-11-04

**Authors:** Yan Wang, Xuesong Zhang, Songhua Xiao, Ning Lu, Zheng Wang, Mi Zhou

**Affiliations:** 1 Department of Orthopaedic Surgery, the PLA General Hospital, Beijing, China

## Abstract

**Objectives:**

To investigate clinical effects and manual operational point of Bryan cervical disc prosthesis in Chinese, to observe the stability and range of movement (ROM) post-operatively.

**Methods and materials:**

From 2003,12 to 2005,12, Bryan disc prosthesis replacement applied in 83 cases (102 levels) of cervical spondylotic myelopathy (CSM) after anterior decompression in our hospital. Clinical (JOA grade and Odom's scale) and radiological (X-ray of flexion, extension; left and right bending position) follow-up was performed. Systemic radiographic study about stability and ROM of replaced level post operationally were measured. CT or MRI scans were applied in all cases to evaluate the signs of the prosthesis deflexion and hetero-ossification in the replaced levels.

**Results:**

At least 12 months follow-up were done in 65/83 of these paients. All of 83 patients were improved according to Odsm's scale. JOA score increased from average 8.7 to 15.5. There was no prosthesis subsidence. Replaced segment achieved stability and restored partial of normal ROM 4.73°(3.7°–5.9°) early postoperation and 8.12°(5.8°–13.6°) more than 12 months postoperation in flex and extension position. No obvious loss of lordosis was found. CT or MRI follow-up shows position deflexion of the prosthesis metal endplates (<1.5 mm) in 14/77 levels and (1.5~3 mm) in 4/77. heter-ossification was found in the replaced levels only in 2 cases.

**Conclusion:**

Byran cervical disc prosthesis restored motion to the level of the intact segment in flexion-extension and lateral bending in post-operative images. At the same time, it can achieve good anterior decompression treatment effect and immediate stability in replaced 1 or 2 levels, and which is a new choice for the treatment of CSM.

## Background

Anterior cervical decompression and fusion have traditionally been considered one of the most successful of cervical spine surgeries; however, the problems associated with cervical fusion have been illuminated recently [1,2]. A reduction in range of motion and increasing stress at adjacent levels are commonly accepted as pitfalls of fusion surgery. Motion in the cervical spine occurs at both the interbody. Fusion techniques typically aim to stop motion at either one or both of these regions. Loss of motion either anteriorly or posteriorly can lead to subsequent ankylosis at the other motion center [3].

Cervical disc replacement became favorable in recent years. Several studies on the effectiveness of Bryan cervical disc prosthesis in treating radiculopathy, spondylotic myelopathy have been conducted in Europe and Australia. But to date, no study on the clinical result of Bryan cervical disc prosthesis in China has been reported. No systematic radiographic study among Chinese about the post operation result on range of motion (ROM) of replaced level or stability of the prosthesis has been reported either. In order to find out the clinical result of CSM of which among Chinese, a prospective but non-random clinical and radiographic study was conducted in our hospital (one of the largest orthopaedic department in China). The main purpose of this study is to find out the treatment result and key points in the operation procedure of Bryan cervical disc prosthesis among Chinese. The post-operation stability and range of motion were also measured.

## Methods

### Patient sample

83 patients (102 levels) with CSM were selected for this study. Detailed neurological examination and JOA (Japanese Orthopaedic Association Scale; JOA) grading were conducted to ensure that all the patients are qualified for anterior cervical decompression in the space level. MRI scan indicates that all patients have spinal cord compression with 1 or 2 segments. No instability or hypermobility was identified by flexion and extension cervical x-rays. The 83 patients were not randomly selected, but as a continuous group. Before the operation, cervical arthroplasty had been explained to all patients, and were chosen by those patients as treatment procedure.

### Study conduction

**Figure 1:**
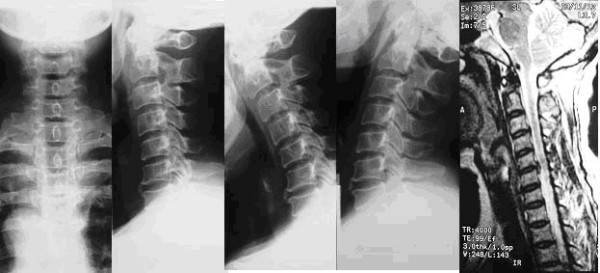
53 y, female, CSM. Preoperative anteroposterior and lateral x-rays show straight alinement and degeneration. Lateral flexion/extension x-rays show the range of motion before operation. And sagittal MRI T2 show that extensive disc degeneration in cervical disc. Hight loss of disc space and spinal cord compression can be found in C5/6.

**Figure 2:**
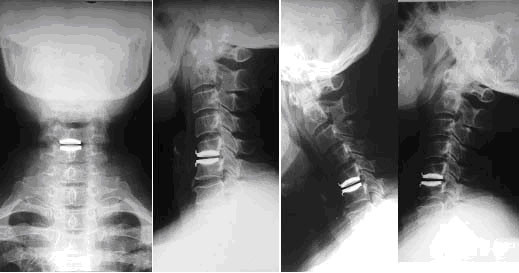
24 months postoperative anteroposterior (A) and lateral (B) x-rays after disc replaced at C5/6, Lateral flexion/extension x-rays(C, D) show restored ROM, and no prosthesis excusion or subsidence.

**Figure 3:**
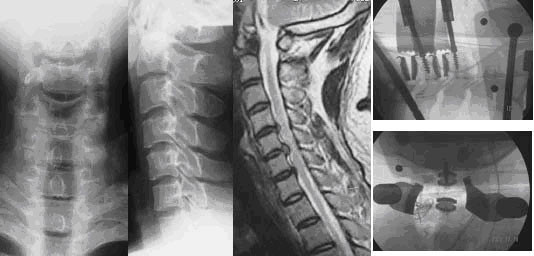
34 y, male, Preoperative anteroposterior and lateral x-rays show loss of the lordosis. And sagittal MRI T2 show that spinal cord compression secondary to disc herniation in C4/5, C5/6. Disc degeneration exists in C6/7, but no anterior compression in coronal plane.

**Figure 4:**
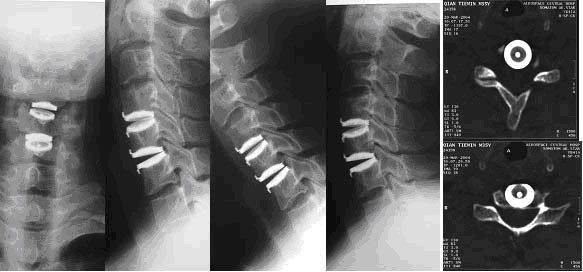
24 months postoperative anteroposterior and lateral x-rays after disc replaced at C4/5,5/6, Lateral flexion/extension x-rays show restored normal ROM. CT scan show no prosthesis excusion or heteostification.

Between December, 2003 and December, 2005, a group of 4 surgeons performed the surgery for 83 patients (102 levels) with CSM. Before each operation, detailed clinical examination and JOA grading were conducted. All of the 83 patients were qualified for the cervical arthroplasty standard presented by Doctor Bryan and Sekhon. 1,3,6,12,24 months follow-up were done in all patients. Among the 83 patients, 65 cases finished at least 12 months follow-up (12 to 28 months). Systematic radiographic study about the stability and ROM of replaced level was performed. Clinical (JOA grade and Odom's scale) and radiological (X-ray of flexion, extension; left and right bending position) were conducted. Computer-Tomographie (CT) or Magnetic Resonance Imaging (MRI) scan were applied to find out the right place of prosthesis and hetero-ossification in the replaced levels (Figures 1, 2, 3, 4).

### Surgical techniques

The patients were placed supine on a fluoroscopic imaging table with their arms at their side. Shoulders taped to allow for fluoroscopic imaging, which were obtained in anterior-posterior and lateral planes to determine the level of diseased disc. and the shoulders were pulled caudally by a heavy bandage (2 kg), when necessary, to secure a clear lateral visualization of the lower cervical spine. A standard right-sided approach was undertaken to access the anterior cervical spine. A set of proprietary surgical retractor system, Medtronic Sofamor Danek, Memphis TN, was used to assist in exposure. After initial discectomy to achieve wide exposure, we used a gravitational referencing system to establish a virtual axis in the intervertebral disc space to help us position a milling fixture. This milling fixture controls the location and movement of the powered cutting instruments for preparing the vertebral endplates that will be replaced by the prosthesis. To our experience, if we keep the angle of tunnel instruments 2~3 degrees more than the measured angle, both the up and lower milled endplates will be exactly parallel to the vertebrae space. This also makes the geometry of the shell's convex outer surface match the milled endplates better, and consequently, the prosthesis is captured inside a ridge of bone. This tight fit provides immediate stability.

### Post-operation patient care

All patients were required to wear hard collar for 1 week (wound healing period), and then freed from assistant support. Clinical and radiological follow-ups were carried out in 1,3,6,12,24 months postoperationly.

## Results

Among these 83 patients, 43 were males, 40 were females. The average age is 48.9 years old (range: 34~58 Yrs). No motor neuron disease was found to any of the patients in this group. The cervical disc prosthesis pattern number is confirmed by CT-scans. (Please see Table 1 for details).

**Table 1:** All of 65 patients (77 levels) were adopted the cervical disc replacement, the static of cervical disc prosthesis in the replaced levels.

level	Patient
C3/4	7
C4/5	19
C5/6	30
C6/7	8
C3/4, C4/5	3
C4/5, C5/6	9
C3/4, C5/6	5
C4/5, C6/7	2

On average, each operation lasted 128 min (range 90–185 min). Neither neurological nor vascular complication was identified during or after the surgeries. According to the Odsm's scale, all of 65 patients (77 levels) followed up at least 12 months improvement (47/65 excellent, 18/65 good). Average JOA score of the 65 patients increased from 8.7 to 15.5 at last follow up. No prosthesis subsidence or excursion was identified. Replaced segment achieved stability and partially restored normal ROM (4.73° (3.7°–5.9°) in 3 months postoperationly and 8.12° (5.8°–13.6°) more than 12 months postoperationly in flexion and extension position, and 3.42° (2.3°–4.4°), 3.16° (2.5°–4.1°) in 3 months postoperationly and 5.08° (3.8°–6.25°), 4.87° (3.2°–7.1°) more than 12 months postoperationly in left and right bending position). No obvious loss of lordosis was found. Result from CT or MRI follow-up shows position deflexion of the prosthesis metal endplates (<1.5 mm) in 14/77 levels and (1.5~3 mm) in 4/77. Heter-ossification was found in the replaced levels only in 2 cases.

### Complication

Complication following insertion may be divided into those specific to the prosthesis and those associated with anterior cervical discectomy. No cerebrospinal fluid leak or wound hematomas in this group, but 1 case of esophageal injury occurred, though 1 week of nasogastric feeding tube and regional drainage, recoverd successfully. No no end plate of Bryan cervical disc migration, 1 case of failure to maintain motion because of spontaneous fusion in replaced segment. No cases of subsidence of device failure. 2 case of worsened cervical regional kyphosis in 9 cases of with preoperative focal kyphosis at operated segments.

## Discussion

Anterior single-level discectomy and/or intervertebral discdecompression and fusion is a common treatment for symptomatic intervertebral cervical spondylosis with myeloradiculopathy., in combination with/without internal fixation is still the most widely accepted treatment for CSM today. This method has significantly improved the treatment result in comparison with the traditional method of posterior decompression. Although it improves pathologic recovery of spinal cord, but fusion leads to cervical ROM loss and increases load to adjacent levels, which maybe will accelerate degeneration of adjacent segments. Hilibrand [4] et al reported that 2.9% patients need additional surgical treatment each year due to complications with adjacent segments after anterior interbody fusion with cervical intervention. Due to the high complication rate and adverse effect on adjacent segments by cervical interbody fusion, a new technique that keeps advantages of anterior decompression while maintaining the impaired motion level for cervical spondylotic disease is required. Cervical disc prosthesis offers a new method for CSM treatment, especially for patients of single level [5]. Such adjacent segment degeneration may be preventable if spinal motion can be maintained by a functional disc prosthesis.

Disc replacement attempts started in the 1950s. In year 1966, Fernstorm [6] tried a method by placing a stainless steel ball into lumber and cervical disc center. Although his attempt did no harm to the patients, there was either no positive treatment effect or the treatment result only lasted for a short period of time. In 1990s, the invention of cervical disc prosthesis makes disc replacement a viable treatment method for CSM disease. Different types of prosthesis, such as Prestige, Bryan, and ProDisc-C were introduced. It is reported that approximately 6,000 patients have been treated with the Bryan disc in Europe and North America until 2005. In 2002, Goffin [7] et al reported for the first time that Bryan Disc (Spinal Dynamics Corp.) was successfully used for treatment of cervical radiculopathy, and achieved 85–90% satisfactory rate in 12 months follow-up. The next year, Sekhon [8] reported that Bryan Disc is used for treating 7 patients with CSM (5/7 single level and 2/7 adjacent two levels). According to Odom's criteria, the result from follow-up between 1 and 17 months after operation shows 5/7 excellent and 2/7 good. All the replaced levels restored motion, but loss of lordosis took place in 4/7. Bryan [9] reported that a total of 97 prosthesis were implanted in patients with single level degenerative disc disease (DDD). Among the 97 patients, 49 were given 12 months follow-up and the result is excellent: 70%, good: 4%, fair: 13%, poor: 13% according to Odom's criteria. 10 patients had finished 24 months follow-up, and similar result was achieved. Gwynedd [10] et al further studied biomechanical profile of the cervical spine following 20 cases of cervical arthroplasty. They found that the overall cervical motion (C2–C7) was moderately but significantly increased during late follow-up. Sagittal rotation, anterior and posterior disc height, translation, and center of motion coordinates did not change significantly following surgery.

The Bryan Cervical Disc prosthesis consists of a low-frition poluurethane nucleus surrounded by a polyurethane sheath and siuated between two titanium alloy shells. The biarticulating metal-on-polymer disc possesses elasticity and little compressibility and allows for unconstrained motion and translation through normal ROM. The prosthesis is axially symmetric, allowing for similar ROM in sagital plane motion and in lateral bending. Biomechanical studies suggest a mobile center of rotation, allowing the device to accommodate a range of preoperative center-of-rotation values without subjecting the facets and ligaments to abnormal stresses [11]. A cohort of 20 patients using quantitative motion analysis software to analyze intervertebral motion, reaching the conclusion that sagittal ROM, centers of rotation, horizontal translation, and disc height were not significantly altered following disc replacemt compared with the preoperative state [12]. So the right place of the prosthesis is important, because that abnormal shifting of the center of rotation following spinal arthroplasty has been implicated in recent reports of facet pain associated with prosthesis positioning[13]. A functional disc prosthesis may be able to restore the functional spine unit and prevent subsequent adjacent segment degeneration.

As described above, cervical arthroplasty has been an alternative to cervical anterior discectomy with fusion. At present, the short-term results are promising [14]. But to date, no study on the clinical result of Bryan cervical disc prosthesis among chinese patients has been reported, and no systemic radiographic study about immediate stability and short-term ROM after the operation has been reported in China either. Does the prosthesis's design (the same hight of 8.5 mm of each size prosthesis) matches anatomic character of Chinese, especially in whose cervical vertebra is small? In 2 cases whose vertebra was small, interspace is also narrow, when inserting the 8.5 mm space enlarger, the lower vertebra leaked, and alignment of posterior corner of vertebra which was in a straight line just adjusted before operation, changed to angled, so the milled track was not as the designed one, Which caused adverse open of prosthesis space. In this group of 83 cases (102 levels), one independent radiologist assessed all radiographs. No subsidence and migration was observed from X-ray photography and no obvious kyphosis was found to any of the patients either. On the contrary, 2 patients, who had cervical kyphosis misalignment before the operation, gained normal cervical lodorsis after disc replacement. In order to be comparable to patient selection in Europe or North America [15,16], only CSM patients with single or adjacent or jump 2 levels were chosen this group. The prosthesis restores height of degenerated disc space and relieves the spasm of cervical muscle. Post-operation Cobb angles for flexion/extension and lateral bending of the function segment unit (FSU) at the implant level indicates improved ROM. At last follow up of those finished at least 12 months follow up, all of 65/83 cases achieved flexion/extension ROM above 8.12 degrees in replaced site, and left/right lateral bending ROM ranged between 5.08 and 4.87 degrees. According to Odom's criteria, the result of those finished at least 12 months follow up after operation of the 65 patients achieved 47/65 excellent and 18/65 good. To our experience, if we increase 2–3 degrees comparing to the measured angle, the position of the prosthesis will be parallel to introvertebra space maybe due to the more curved lower endplate of cervical vertebra among patients in most Chinese. This finding requires additional anatomic study on cervical vertebra in Chinese to confirm. Result from CT or MRI shows that 14/77 levels with deflexion of prosthesis less than 1.5 mm and 4/77 level with deflexion of prosthesis between 1.5 mm and 3 mm, nonsteonotid drug were not taken as routine postoperatively in this group. Ossification in the replaced levels was identified just in 2 cases. Rotation of cervical alignment in the operation bed during operation is the potential reason for excursion of prosthesis.

Choosing appropriate prosthesis size and insertion position is critical to the success of the operation. Details in these procedures will not only affect instant stability of prosthesis but also alter the axis of rotation and load sharing [17-19]. Surgeon need to make adjustments according to variations in anatomic structure and degenerative conditions in different patients. A potential contraindication of the cervical arthroplasty with metal endplates is osteoporosis which can potentially increase the risk of endplate subsidence into the vertebral bodies [20]. The long-term impact of bone loss with aging due to subsidence of metal endplates requires additional studies. A forward long-term study with multi-center, randomly selected patients among patients in Asia is to be carried out in our hospital.

## Conclusion

CT and MRI images more than 24 months post operation indicate Byran cervical disc prosthesis partly restored motion of the impaired segments in flexion-extension and lateral bending while maintaining the advantage of anterior decompression. Selecting appropriate prosthesis size and insertion position is critical to treatment result. In summary, cervical arthroplaty of Bryan disc can achieve good anterior decompression treatment effect and stability in replaced 1 or 2 levels, which makes it a new choice for the treatment of CSM in Chinese.

## References

[B1] Frank MP, Steven RG (2005). Cervical Disc Replacement. Spine.

[B2] Pickett GE, Sekhon LH, Sears WR, Duggal N (2006). Complications with cervical arthroplasty. J Neurosurg Spine.

[B3] Lali HS, Sekhon (2005). Reversal of Anterior Cervical Fusion with a Cervical Arthroplasty Prosthesis. J Spinal Disord Tech.

[B4] Hilibrand AS, Carlson GD, Palumbo MA, Jones PK, Bohlman HH (1999). Radiculopathy and myelopathy at segments adjacent to the site of a previous anterior cervical arthrodesis. J Bone Joint Surg (Am).

[B5] Mayer HM, Wiechert K, Korge A, Qose I (2002). Minimally invasive total disc replacement: Surgical technique and preliminary clinical results. Eur Spine J.

[B6] Fernstrom U (1966). Arthroplasty with intercorporal endoprosthesis in herniated disc and in painful disc. Acta Chir Scand.

[B7] Goffin Proceedings of the Annual Meeting of the Spinal Arthroplasty Society, May 2002, Montpellier, France.

[B8] Sekhon LH (2003). Cervical arthroplasty in the management of spondylotic myelopathy. J Spinal Disord Tech.

[B9] Bryan VE (2002). Cervical motion segment replacement. Eur Spine J.

[B10] Azmi H, Schlenk RP (2003). Surgery for postarthrodesis adjacent-cervical segment degeneration. Neurosurg Focus.

[B11] Wigfield C, Gill S, Nelson R, Langdon I, Metcalf N, Pobertson J (2002). Influence of an artificial cervical joint compared with fusion on adjacent-level motion in the treatment of degenerative cervical disc disease. J Neurosurg.

[B12] Cunningham BW, Gordon JD, Dmitriev AE, Hu N, Mcafee PC (2003). Biomechanical evaluation of total disc replacement arthroplasty: an in vitro human cadaveric model. Spine.

[B13] Gwynedd EP, Jeffrey PR, Neil D (2005). Kinematic: Analysis of the Cervical Spine Following Implantation of an Artificial Cervical Disc. Spine.

[B14] John BP, Vincent CT (2005). Treatment of the Painful Motion Segment: Cervical Arthroplasty. Spine.

[B15] Wigfield CC, Gill SS, Nelson RJ, Metcalf NH, Roberson JT (2002). The new Frenchay artificial cervical joint: results from a two-year pilot study. Spine.

[B16] Cummings BH, Robertson JT, Gill SS (1998). Surgical experience with an implanted artificial cervical joint. J Neurosurg.

[B17] Wiffield CC, Skrzypiec D, Jackowski A, Adams MA (2003). Internal stress distribution in cervical intervertebral discs: the influence of an artificial cervical joint and simulated anterior interbody fusion. J Spinal Disord Tech.

[B18] DiAngelo DJ, Roberston JT, Metcalf NH, McVay BJ, Davis RC (2003). Biomechanical testing of an artificial cervical joint and an anterior cervical plate. J Spinal Disord Tech.

[B19] Pointillart V (2001). Cervical disc prosthesis in humans: First failures. Spine.

[B20] Scott DB, Richard AB, John GH, Hanley EN, Zigler JE (2004). Disc Replacements: This Time Will We Really Cure Low-Back and Neck Pain?. J Bone Joint Surg (Am).

